# Family History of Hypertension Predicts Thyroid Cancer Risk in Women: A Population‐Based Cross‐Sectional Study With Integrative Machine Learning and Genomic Analyses

**DOI:** 10.1002/cam4.71031

**Published:** 2025-08-10

**Authors:** Zijin Wang, Yanhui Lin, Fanke Meng, Yuxin Sun, Ziran Zhang, Tong Wu, Min Fu, Fanye Wu, Zhengran Li, Zejun Chen

**Affiliations:** ^1^ The Second Clinical Medicine School Southern Medical University Guangzhou Guangdong China; ^2^ Health Management Center, The Third Xiangya Hospital Central South University Changsha China; ^3^ Emergency Department Zhujiang Hospital of Southern Medical University Guangzhou Guangdong China; ^4^ The First Clinical Medicine School Southern Medical University Guangzhou Guangdong China; ^5^ Department of Ophthalmology, Zhujiang Hospital Southern Medical University Guangzhou Guangdong China

**Keywords:** biomarkers, epidemiology, genomics, risk model, TCGA, thyroid cancer

## Abstract

**Introduction:**

Thyroid cancer is one of the most common endocrine malignancies globally, with a markedly higher incidence in women. Although pregnancy‐induced hypertension is recognized as a risk factor, the underlying mechanisms linking hypertension and thyroid cancer remain poorly understood. This study explores the gender‐specific associations between family history of hypertension and thyroid cancer, integrating clinical characteristics with genetic insights.

**Methods:**

In this large‐scale cross‐sectional study conducted in China, clinical and lifestyle data were collected from 52,963 participants, including 296 thyroid cancer cases. An interpretable ensemble machine learning model was constructed to evaluate risk factors, and odds ratios (ORs) with 95% confidence intervals (CIs) were calculated. Additionally, the Cox proportional hazards model was applied to identify significant hypertension‐related genes, and Kaplan–Meier analyses were used to compare overall survival, clinical stages, and immune cell infiltration between high‐ and low‐risk groups.

**Results:**

Our analysis revealed that individuals with a family history of hypertension exhibited a significantly altered rate of thyroid cancer (*p* < 0.001). In particular, among women, a positive family history increased thyroid cancer risk (OR = 1.53, 95% CI: 1.09–2.14, *p* = 0.04) and emerged as a key predictor in the machine learning model. Genetic analyses identified overlapping genes—most notably FOXD3, F10, and SLC12A5—whose aberrant expression was associated with poorer five‐year survival and distinct immune cell profiles.

**Conclusions:**

Our study provides novel clinical and genetic evidence that family history of hypertension is significantly associated with thyroid cancer in women. Integrating hypertension‐related screening with genetic profiling may enhance risk stratification and aid in the development of personalized management strategies to reduce overtreatment.

## 1 Introduction

Thyroid cancer is among the most common endocrine malignancies, and its incidence is rising annually [1, 2]. The Global Cancer Statistics Survey 2020 reported 586,000 cases of thyroid cancer worldwide, ranking ninth in incidence and accounting for 3% of all cancers [3]. Worse still, women are far more likely to develop thyroid cancer than men, which is three times more common in women than in men. Thyroid cancer is the fourth most common cancer in women, with an age‐standardized mortality rate (ASMR) of 0.5/100,000 in 2020 [4]. In China, there were 3,002,899 cancer‐related deaths in 2020, representing 30.2% of all cancer deaths globally, with thyroid cancer ranking seventh in age‐standardized incidence rate (ASIR) [5].

Some studies have reported that factors influencing thyroid cancer include sex, family history of thyroid cancer, body mass index (BMI), smoking [6, 7], alcohol consumption [8], household income [9], physical activities [10, 11], reproductive factors, diet, and sleep [12, 13, 14]. The widespread use of ultrasound and other modern diagnostic techniques has enabled detection of previously undetectable thyroid cancers. However, recent studies, particularly in China, have highlighted the issue of overtreatment due to the detection of indolent thyroid cancers [15, 16, 17]. Therefore, it is crucial for researchers to thoroughly examine the factors associated with thyroid cancer risk and to accurately predict its prognosis.

Hypertension is the leading modifiable risk factor for cardiovascular (CV) disease, a major contributor to global mortality and morbidity, and is the primary cause of death in China [18, 19, 20]. Some studies have reported differences in the prevalence of hypertension between men and women. Although premenopausal women have a lower prevalence and severity of hypertension, and therefore CV, the risk increases dramatically after menopause [21, 22]. Moreover, women tend to respond less favorably to hypertension treatment and face a higher risk of death following a cardiovascular event [23]. A nationwide survey of 451,755 participants across 31 provinces in China found that 23.2% (approximately 244.5 million) of Chinese adults had hypertension, with significant differences observed between men and women in all characteristics except ethnicity [24]. Previous studies have established a correlation between gestational hypertension and thyroid cancer [25], and emerging evidence suggests that hypertension may be intrinsically linked to thyroid cancer development [26, 27].

In this large cross‐sectional study, we investigated the relationship between thyroid cancer and family history of hypertension and evaluated the predictive role of this family history using an interpretable integrated model. In addition, we examined the genetic link between thyroid cancer and hypertension to assess its prognostic impact in women.

## 2 Materials and Methods

### 2.1 Demographic and Clinical Characteristics

Data for this cross‐sectional study were obtained from the Third Xiangya Hospital of Central South University between 2020 and 2021 (*n* = 103,649). After removing duplicates, incomplete records, abnormal entries, and data that did not meet the minimum criteria (*n* = 50,586), 52,963 subjects (296 patients with thyroid cancer) were included in the analysis. Written informed consent was obtained from all participants, and the study protocol was approved by the Ethics Committee of the Third Xiangya Hospital of Central South University (No. 22206). The study was conducted in accordance with the principles set forth in the Declaration of Helsinki.

For baseline data acquisition, all participants completed a nationally standardized medical examination questionnaire commonly used in Chinese health‐screening centers. The questionnaire collected basic demographic information, lifestyle factors, psychological status, personal medical history, and family medical history. Family history of hypertension was defined as a record of one or more first‐degree relatives with a high blood pressure.

### 2.2 Selection of Clinical Features Related to Thyroid Cancer

The collected psychological factors were quantified using a standardized scale, and their reliability was evaluated using Cronbach's alpha coefficient (Table S1). Clinical data from the physical examination survey were stratified by sex. Continuous variables were compared using analysis of variance (ANOVA) and the Kruskal–Wallis test, with results expressed as mean ± standard deviation (SD). Categorical variables were assessed using chi‐square tests, with outcomes presented as frequencies. Subsequently, binary logistic regression was performed to incrementally identify eligible factors using forward, backward, and bidirectional feature selection methods and to calculate their odds ratios (OR) and confidence intervals (CIs).

### 2.3 Construction of Explainable Prediction Models

To address the class imbalance between positive and negative samples, the synthetic minority oversampling technique (SMOTE) was applied to positive samples, increasing them by a factor of ten. The negative samples were then split into ten subsets, each of which was merged with the oversampled positive samples in turn. To observe the structure of the data before and after amplification more clearly, we compared the results before and after data amplification (Table S2). XGBoost, decision trees, gradient boosting decision trees (GBDT), CatBoost, and logistic regression were used to build individual models for every subset, and five integrated models were subsequently constructed using soft voting. In this soft voting mechanism, each of the ten constituent sub‐models was assigned an equal weight when aggregating their probabilistic predictions, consistent with the default implementation of the VotingClassifier when specific weights are not provided. The optimal model was selected based on the area under the receiver operating characteristic curve (AUC). Furthermore, SHapley additive explanations (SHAP) were employed to analyze the feature importance in the integrated model.

For each of the five base machine learning algorithms employed, a specific grid of hyperparameters was pre‐defined. The process of hyperparameter optimization for each of the ten sub‐models (developed for each algorithm type, corresponding to the ten balanced data subsets) involved iterating through these pre‐defined parameter combinations using a grid search approach. The optimal set of hyperparameters for each sub‐model was determined by its performance, specifically the AUC, on an independent validation set. The hyperparameter search grids for each model type are detailed in Table S3.

### 2.4 Acquisition of Relevant Genetic Data

Regarding the genetic database, transcriptome profiles of thyroid cancer patients were downloaded from The Cancer Genome Atlas (TCGA) (https://portal.gdc.cancer.gov/) on August 21, 2022. To ensure cohort homogeneity for genetic analysis, we specifically selected female patients diagnosed with papillary thyroid cancer (PTC). This resulted in a final dataset of 414 tissue samples used for our genomic analyses, comprising 368 PTC tumor samples and 46 normal thyroid tissue samples from female individuals. The TCGA female PTC cohort utilized for these genomic analyses was ethnically diverse, consisting of 246 White, 38 Asian, 21 Black or African American individuals, with the remainder belonging to other ethnic groups or having unreported ethnicity. In the current study, no specific ethnic stratification or statistical adjustment for ethnicity was performed during the TCGA genomic data analyses, primarily due to the limited sample sizes within some of the non‐White subgroups, which would reduce the statistical power for robust ethnicity‐specific subanalyses. For comparison, our primary clinical study cohort, from which lifestyle and clinical data were collected, was recruited in China and is predominantly of Chinese ethnicity.

TIMER (https://cistrome.shinyapps.io/timer/) is a web server designed for comprehensive analysis of tumor‐infiltrating immune cells (TIICs). We employed the “Gene” module to evaluate the relationship between the expression of selected genes and the infiltration levels of six immune cell types: dendritic cells, macrophages, neutrophils, CD8+ T cells, CD4+ T cells, and B cells.

### 2.5 Overlapping Expression Gene Selection

Transcriptome profiles, specifically raw gene counts (unstranded type as per GDC file specifications), for the TCGA female PTC cohort were downloaded from the Genomic Data Commons (GDC) data portal and subsequently processed to generate a unified gene count matrix where rows represent gene symbols and columns represent individual samples. Subsequent differential gene expression analysis between tumor and normal samples was performed using the DESeq2 R package. The statistical model for DESeq2 was specified with the design formula ~ condition, where the “condition” variable denoted the biological sample type. It is acknowledged that large‐scale datasets such as TCGA, despite undergoing standardized processing by the consortium, can be susceptible to technical variations, often referred to as batch effects, arising from factors like different processing plates or tissue source sites. In the current iteration of our genomic analysis pipeline for identifying differentially expressed genes, explicit batch effect correction steps, such as the use of ComBat or the inclusion of known batch covariates in the DESeq2 design formula, were not implemented prior to or during the differential expression analysis reported herein. The DESeq2 normalization procedure was applied as part of its standard workflow based on the specified design.

We identified differentially expressed genes in thyroid cancer using RNA‐seq data from female patients with thyroid cancer in the TCGA database. The hypertension‐related genes were retrieved from the OMIM, GEO, and GeneCards databases to construct overlapping gene sets. These overlapping genes were then cross‐compared with the previously identified differentially expressed thyroid cancer genes, and significantly expressed overlapping genes (adjusted *p* < 0.05; |log_2_FoldChange| > 2) were selected.

### 2.6 Prognostic Analysis of Expressed Genes

**FIGURE 1:**
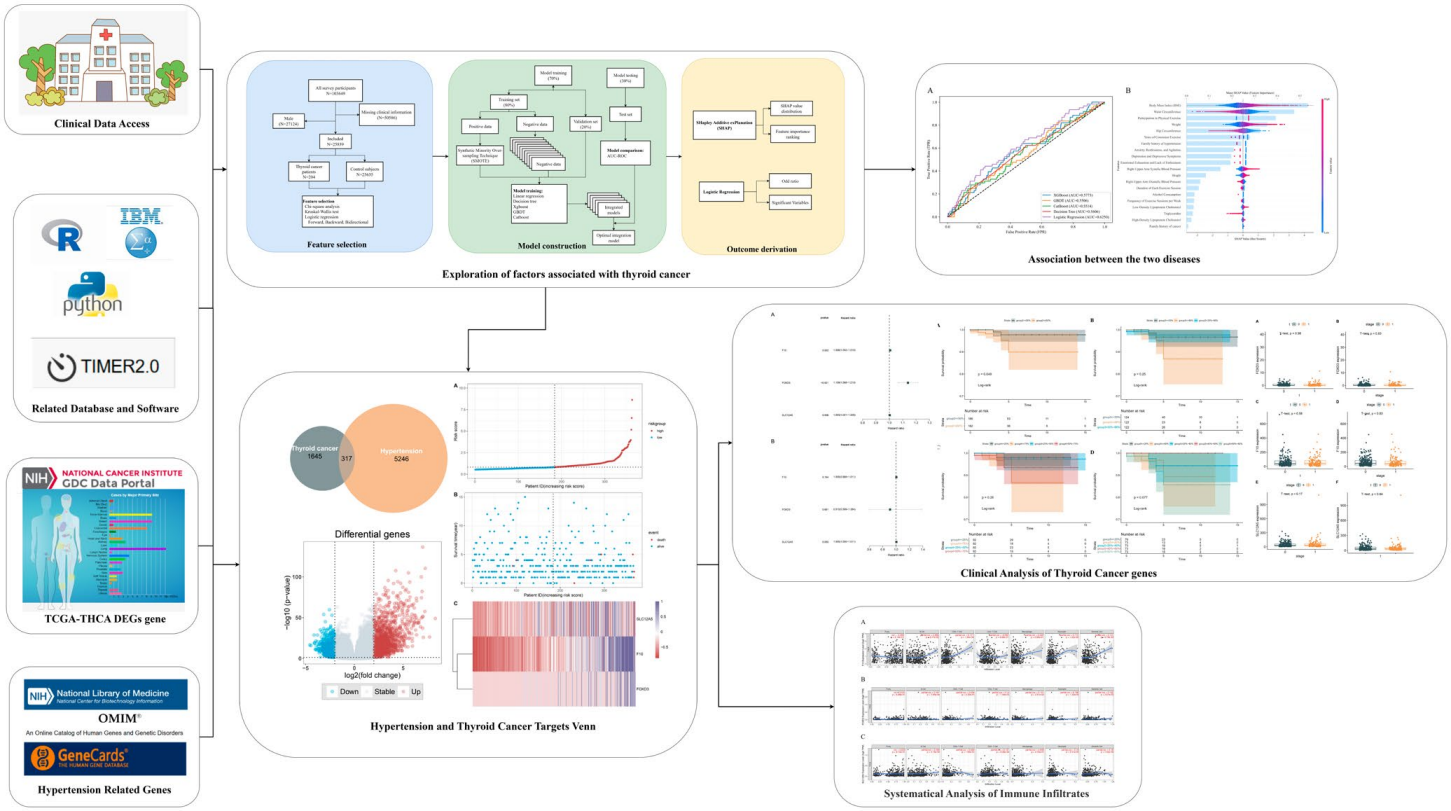
Flowchart of the research on the association between thyroid cancer and hypertension family history.

In terms of gene prognostic analysis, we first identified significantly associated genes using univariate and multivariate Cox proportional hazards regressions. Based on these findings, we examined the genetic prevalence and clinical characteristics of patients with thyroid cancer. Patients were divided into low‐ and high‐risk groups according to a median gene‐related risk score, and Kaplan–Meier survival curves were generated using the log‐rank test to compare the two groups. We further analyzed the prognosis and clinical correlation of individual genes in both groups according to the stage and T stage. In addition, we employed the TIMER online tool for immune cell correlation analysis to gain further insight into the role of these genes in thyroid cancer progression and immune cell infiltration (Figure 1).

### 2.7 Statistical Analysis

All analyses were conducted using R Studio (version 4.1.2) and Python (version 3.10). Statistical significance was defined as *p* < 0.05, with all *p* values reported as two‐sided.

## 3 Result

### 3.1 Descriptive Analysis of Clinical Data

This large cross‐sectional study enrolled 296 patients with thyroid cancer, including 92 males and 204 females. Table 1 presents the baseline characteristics of the participants with and without thyroid cancer. A notable sex difference was observed as the incidence of thyroid cancer was substantially higher in women than in men. In terms of family history, the proportion of participants with thyroid cancer who also had a family history of cancer was significantly higher (*p* < 0.001) than those without thyroid cancer. Smoking was inversely associated with thyroid cancer (*p* < 0.001). Similarly, various measures of alcohol consumption, including frequency, total amount, duration, and type, were negatively correlated with the incidence of thyroid cancer (*p* < 0.001).

**TABLE 1:** Demographic and clinical characteristics of participants.

Characteristic	Patients with thyroid cancer (*n* = 296) (*n*, %)	Controls (*n* = 52,767) (*n*, %)	*p*
Age (years)			
Mean (SD) [range]	42.31 (10.65) [25–76]	42.52 (12.35) [13–95]	0.832
Median (IQR)	41 (16)	40 (18)	
Sex			
Male	92 (31.08)	27,132 (51.42)	< 0.001
Female	204 (68.92)	25,635 (48.58)	
Family history			
Other	103 (34.8)	27,534 (52.18)	< 0.001
Hypertension	93 (31.42)	15,236 (28.87)	
Diabetes mellitus	26 (8.78)	4147 (7.86)	
Coronary heart disease	13 (4.39)	1656 (3.14)	
Cancer	61 (20.61)	4194 (7.95)	
Smoking status			
Never smokers	255 (86.15)	38,736 (73.41)	< 0.001
Passive	6 (2.03)	2164 (4.1)	
Former	12 (4.05)	1543 (2.92)	
Active	23 (7.77)	10,324 (19.57)	
Alcohol consumption			
Never drinkers	260 (87.84)	38,250 (72.49)	< 0.001
Active	30 (10.13)	13,868 (26.28)	
Former	6 (2.03)	649 (1.23)	
Type of wine			
Never drinkers	260 (87.837)	38,250 (72.49)	< 0.001
Chinese Baijiu	14 (4.73)	6048 (11.46)	
Beer	5 (1.689)	4229 (8.02)	
Red wine	14 (4.73)	3379 (6.4)	
Other	3 (1.014)	861 (1.63)	
Drinking intensity (drinks/week)			
Never drinkers	260 (87.837)	38,250 (72.489)	< 0.001
1–2	30 (10.13)	10,881 (20.621)	
3–5	4 (1.351)	2777 (5.263)	
> 5	2 (0.676)	859 (1.628)	
Alcohol consumption (liters/drink)			
Never drinkers	260 (87.837)	38,250 (72.489)	< 0.001
< 0.1	16 (5.4)	6976 (13.22)	
0.1–0.25	15 (5.068)	5919 (11.217)	
> 0.25	4 (1.351)	1064 (2.016)	
Unknown	1 (0.338)	558 (1.058)	
Drinking duration (years)			
Never drinkers	260 (87.837)	38,250 (72.489)	< 0.001
< 5	7 (2.365)	3758 (7.121)	
5–10	10 (3.379)	4433 (8.401)	
10–20	12 (4.054)	3533 (6.695)	
> 20	7 (2.36)	2793 (5.293)	
Frequent late‐night snacking (times/week)			
No	213 (71.96)	32,038 (60.7)	< 0.001
= 1	80 (27.03)	19,050 (36.1)	
> 1	3 (1.01)	1679 (3.2)	
Binge eating			
No	286 (96.62)	50,332 (95.39)	0.402
Yes	10 (3.38)	2435 (4.61)	
Staple food types			
Fine grains mainly	108 (36.49)	21,336 (40.43)	0.039
Coarse and fine grains	145 (48.99)	21,714 (41.15)	
Coarse grains mainly	10 (3.38)	2750 (5.21)	
Other	33 (11.15)	6967 (13.2)	
Sleep quality			
Poor	23 (7.77)	4026 (7.63)	0.768
Fair	144 (48.65)	26,772 (50.74)	
Good	129 (43.58)	21,969 (41.63)	
Sleep duration (hours/day)			
< 5	15 (5.07)	3708 (7)	0.156
5–7	204 (68.92)	34,096 (64.6)	
7–9	73 (24.66)	14,596 (27.7)	
> 9	4 (1.35)	367 (0.7)	
Exercise status			
Seldom	89 (30.07)	19,451 (36.9)	0.016
Frequently	207 (69.93)	33,316 (63.1)	
Exercise intensity^a^			
No	89 (30.067)	19,451 (36.862)	< 0.001
Low	98 (33.109)	14,917 (28.27)	
Moderate	54 (18.243)	6922 (13.118)	
High	55 (18.581)	11,477 (21.751)	
Exercise frequency (times/week)			
No	89 (30.07)	19,451 (36.86)	0.042
1–2	81 (27.36)	14,672 (27.81)	
3–5	85 (28.72)	12,878 (24.41)	
> 5	41 (13.85)	5766 (10.93)	
Exercise duration (minutes/day)			
No	89 (30.07)	19,451 (36.86)	0.014
< 30	41 (13.85)	8715 (16.52)	
30–60	129 (43.58)	19,157 (36.3)	
> 60	37 (12.5)	5444 (10.32)	
Exercise history (years)			
No	89 (30.07)	19,451 (36.86)	0.006
< 1	59 (19.93)	8657 (16.41)	
1–5	99 (33.45)	13,728 (26.02)	
6–10	21 (7.09)	4546 (8.62)	
> 10	28 (9.46)	6385 (12.1)	
BMI^b^			
< 18.5	8 (2.7)	1822 (3.45)	0.728
18.5 ~ 23.9	156 (52.7)	26,909 (51)	
24 ~ 27.9	97 (32.77)	18,358 (34.79)	
> 27.9	35 (11.82)	5678 (10.76)	
Height (cm)			
Mean (SD) [range]	162.68 (7.26) [145.50–184.50]	163.97 (8.08) [133.50–205.00]	0.003
Median (IQR)	161.50 (9.50)	163.50 (12.00)	
Weight (kg)			
Mean (SD) [range]	64.06 (12.50) [41.50–111.70]	64.50 (12.30) [31.90–140.40]	0.284
Median (IQR)	61.50 (16.38)	63.10 (17.70)	
Waist circumference^b^ (cm)			
Mean (SD) [range]	79.67 (10.96) [57–123]	80.76 (10.65) [51–135]	0.022
Median (IQR)	77.5 (15)	80 (16)	
SBP^b^ (MmHg)			
Mean (SD) [range]	119.97 (13.26) [90–161]	121.21 (15.22) [67–214]	0.477
Median (IQR)	120.5 (19.75)	120 (21)	
DBP^b^ (MmHg)			
Mean (SD) [range]	73.36 (9.99) [52–107]	73.73 (10.9) [41–136]	0.760
Median (IQR)	73 (13)	73 (15)	
Hip circumference^b^, ^c^ (cm)			
Mean (SD) [range]	94.89 (6.26) [81–120]	94.6 (5.96) [63–132]	0.605
Median (IQR)	94 (7.75)	94 (8)	
Triglycerides^d^ (Mmol/L)			
Mean (SD) [range]	1.53 (1.16) [0.22–8.55]	1.73 (1.71) [0.19–30.84]	0.039
Median (IQR)	1.195 (1.04)	1.27 (1.14)	
HDL‐C^d^ (Mmol/L)			
Mean (SD) [range]	1.34 (0.26) [0.79–2.45]	1.31 (0.29) [0.2–3.33]	0.040
Median (IQR)	1.33 (0.3375)	1.28 (0.38)	
LDL‐C^d^ (Mmol/L)			
Mean (SD) [range]	2.88 (0.79) [0.88–5.2]	2.93 (0.81) [0.03–10.83]	0.434
Median (IQR)	2.89 (0.9625)	2.88 (1.04)	
FPG^d^ (Mmol/L)			
Mean (SD) [range]	5.53 (0.9) [3.25–12.5]	5.58 (1.17) [2.86–23.33]	0.515
Median (IQR)	5.395 (0.76)	5.36 (0.7)	

Abbreviations: BMI, body mass index; DBP, diastolic blood pressure; FPG, fasting plasma glucose; HDL‐C, high density lipoprotein cholesterol; IQR, interquartile range; LDL‐C, low density lipoprotein cholesterol; SBP, systolic blood pressure; SD, standard deviation.

^a^ Different exercise was classified according to its intensity. (low intensity: walking, stair climbing, Tai Chi, yoga, other; moderate intensity: jogging, ballroom dancing, aerobics; high intensity: swimming, ball games, strength exercise, hiking, running, cycling).

^b^ Clinical measurements include physical measurements as well as blood measurements. Physical measurements include height, weight, waist circumference, hip circumference, right upper arm systolic blood pressure, and right upper arm diastolic blood pressure, all measured by experienced health care professionals according to standard protocols. Body mass index (BMI) is calculated from height and weight (kg/m^2^).

^c^ As there were two pregnant women among the participants, these two cases were excluded from the calculation of waist circumference.

^d^ Blood measurements were taken by collecting fasting blood samples after an overnight fast to provide triglycerides, HDL‐C, LDL‐C, and FPG.

Patients with thyroid cancer had lower height (*p* = 0.003), waist circumference (*p* = 0.022), and triglyceride levels (*p* = 0.039) than those without thyroid cancer, whereas HDL‐C levels were relatively higher (*p* = 0.040). Furthermore, an analysis restricted to female participants revealed that compared with women without thyroid cancer, women with thyroid cancer showed a higher proportion of family history of hypertension (Table S4).

Binary logistic regression analysis among the female participants showed that family history of hypertension was associated with thyroid cancer (*p* = 0.04). Notably, the proportion of female thyroid cancer patients with a family history of hypertension was significantly higher than that of those without such a history (OR = 1.53, 95% CI: 1.09–2.14). Women with a family history of cancer exhibited a significantly elevated risk of thyroid cancer (OR = 3.0, 95% CI: 2.06–4.35, *p* < 0.001). Alcohol consumption was also associated with a lower risk of thyroid cancer (OR = 0.41, 95% CI: 0.18–0.92; *p* = 0.03) (Table 2).

**TABLE 2:** Binary logistic regression results for women.

Characteristic	Female with thyroid cancer (*n* = 204), (*n*, %)	Controls (*n* = 25,635), (*n*, %)	OR (95% CI)	*p*
Family history				
Other	94 (46.08)	15,457 (60.3)	1 [Reference]	NA
Hypertension	70 (34.31)	8001 (31.21)	1.53 (1.09–2.14)	0.04
Cancer	40 (19.61)	2177 (8.49)	3.0 (2.06–4.35)	< 0.001
Alcohol consumption				
Never or Former	198 (97.06)	23,977 (93.53)	1 [Reference]	NA
Active	6 (2.94)	1658 (6.47)	0.41 (0.18–0.92)	0.03
Depressed and frustrated				
Never	156 (76.47)	17,645 (68.83)	1 [Reference]	NA
Sometimes	39 (19.12)	6927 (27.02)	0.66 (0.46–0.94)	0.02
Always	9 (4.41)	1063 (4.15)	NA	> 0.05
Weight			1.07 (1.04–1.10)	< 0.001
Mean (SD) [range]	58.51 (8.32), [41.50–102.0]	56.23 (7.87), [31.90–119.0]		
Median (IQR)	58.00 (52.88–62.75)	55.30 (50.90–60.50)		
Exercise Duration (hours)			1.24 (1.09–1.42)	0.002
Mean (SD) [range]	1.33 (1.04), [0.0–3.0]	1.09 (1.03), [0.0–3.0]		
Median (IQR)	2.0 (0.0–2.0)	1.00 (0.0–2.0)		
Waist Circumference			0.96 (0.93–0.99)	0.005
Mean (SD) [range]	75.01 (7.97), [57.0–113.0]	74.11 (8.16) [52.0–128.0]		
Median (IQR)	74.0 (69.0–79.0)	73.0 (68.0–79.0)		

Abbreviations: CI, confidence interval; IQR, interquartile range; OR, odds ratio; SD, standard deviation.

To obtain more detailed information regarding the risk factors, we performed forward and backward feature selection based on the original bidirectional screening. In addition to the factors already identified, high‐density lipoprotein cholesterol, low‐density lipoprotein cholesterol, total cholesterol, triglycerides, and several psychosocial factors showed significant differences between healthy participants and those with thyroid cancer (Table S5).

In the binary logistic regression results for men, we found that although thyroid cancer patients had a markedly higher prevalence of family history of cancer than men without thyroid cancer (OR = 4.35, 95% CI: 2.52–7.50, *p* < 0.001), no comparable association was observed for family history of hypertension (*p* = 0.032) (Table S6).

### 3.2 Interpretation of the Prediction Model Results

**FIGURE 2:**
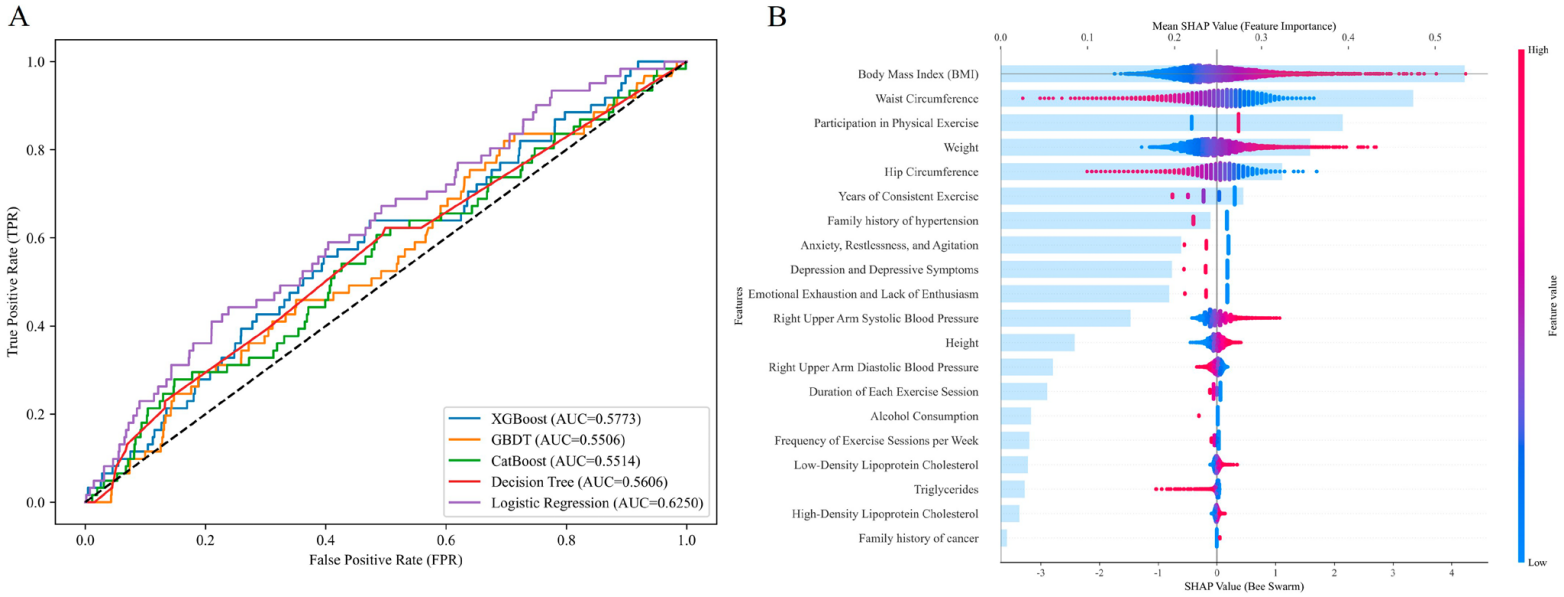
Construction and interpretation of integrated models (A) Receiver operating characteristic curves of five integrated models. It can reflect the accuracy of different models to some extent. It can be found that among all the integrated models, the linear regression model performs best. (B) SHapley Additive exPlanations for integrated model feature variables. It is found that family history of hypertension is an important characteristic variable in the linear optimal model, ranking relatively high.

In this study, we combined the differential variables identified through binary logistic regression, chi‐square analysis, and the Kruskal–Wallis test and applied covariance analysis to select variables to construct an integrated model. The results showed that the binary logistic regression synthesis model outperformed the other four machine learning models in the test set (AUC = 0.625) and exhibited substantial improvement compared with the 10 sub‐models. Moreover, the SHAP analysis indicated that family history of hypertension played a more prominent role in predicting thyroid cancer incidence among women and was positively associated with the disease. Additionally, family history of hypertension appeared to have a stronger internal association with the incidence of thyroid cancer in women than other factors (e.g., psychological factors, smoking, and alcohol consumption) (Figure 2B).

### 3.3 Analysis of Overlapping Genes

**FIGURE 3:**
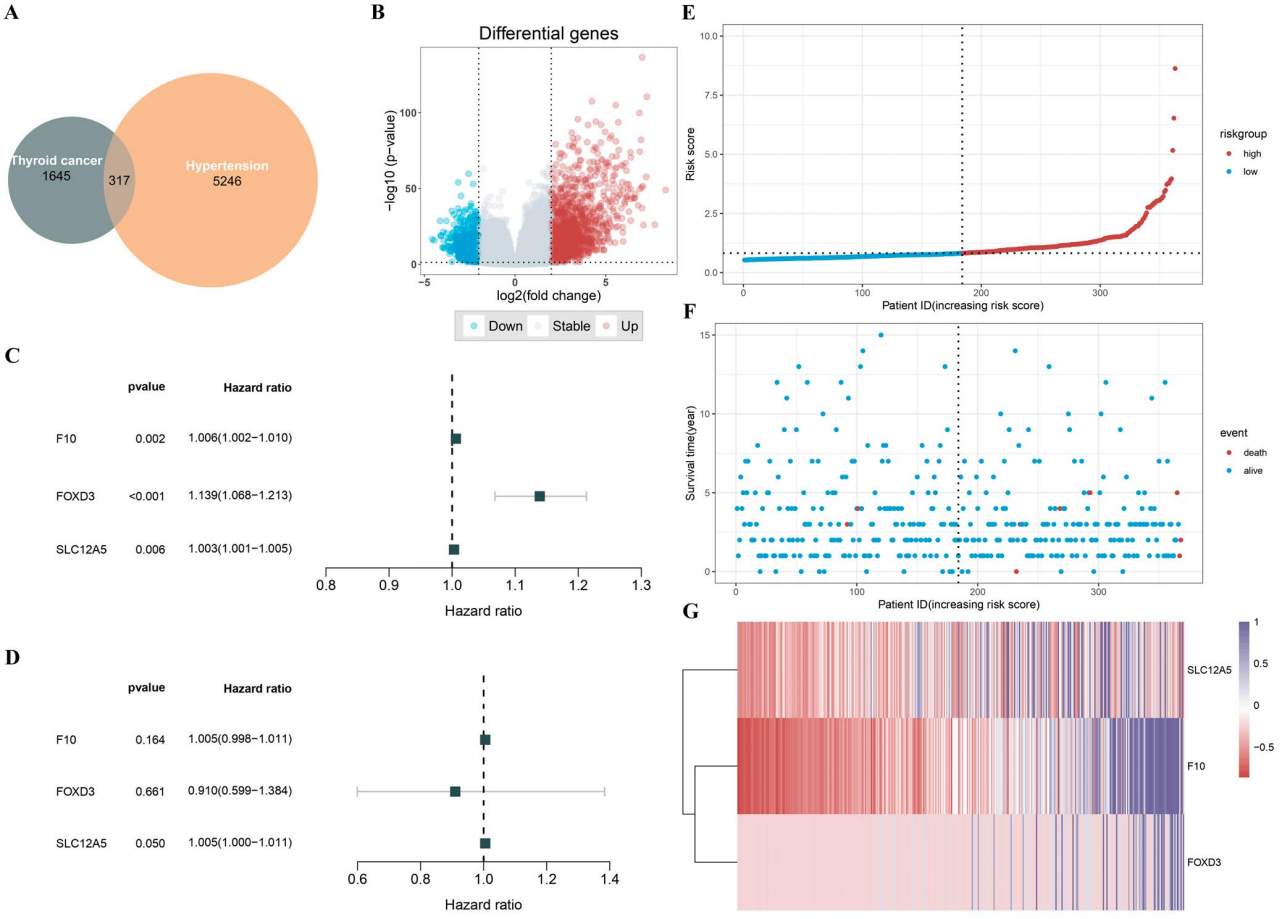
Exploration of genes related to hypertension and thyroid cancer in women (A) Volcano map of differential genes in thyroid cancer. (B) Venn diagram of overlapping differential genes. (C) Forest plot of multivariate Cox proportional‐hazards regression models associated with male genetics. Multivariate Cox proportional‐hazards regression analysis identified three genes, including F10, FOXD3, and SLC12A5. (D) Forest plot of univariate Cox proportional‐hazards models associated with male genetics. (E) Analysis of patients' risk scores using Cox proportional risk regression from the median grouping into high/low‐risk group. (F) Response to survival time of different subjects (G) Heat maps of related genes.

A total of 5563 genes genetically associated with hypertension were identified from the Genecard, OMIM, and NCBI databases. Differential gene expression (DGE) of the intersecting genes was analyzed using the TCGA database. A comparison of these two gene clusters identified 317 overlapping genes related to both hypertension genetics and thyroid cancer, with 226 genes being upregulated and 91 being downregulated (Figure 3A,B). Upon further quality checks, we found that the GC lacked appropriate controls and, therefore, removed GC‐related data from the expression dataset.

### 3.4 Prognostic Expression of Risk Genes

Univariate and multivariate Cox proportional hazard regression analyses were performed on the remaining 316 differentially expressed genes, revealing that 41 of these genes were significantly associated with both thyroid cancer and hypertension (*p* < 0.05) (Table S7). From this group, three genes, F10, FOXD3, and SLC12A5, were selected based on the significance of their univariate analysis results (*p* < 0.01) (Figure 3C). Hazard ratio analysis indicated that elevated F10 (HR = 1.006, *p* < 0.002), FOXD3 (HR = 1.14, *p* < 0.001), and SLC12A5 (HR = 1.003, *p* < 0.05) expression in patients with thyroid cancer was closely associated with poorer prognoses.

Furthermore, we extracted expression data for F10, FOXD3, and SLC12A5 from a male‐specific gene database (Figure 3D). Among males, only SLC12A5 showed a significant difference (*p* = 0.05). These findings suggest that FOXD3 may be associated with hypertension and increased incidence of thyroid cancer in women.

### 3.5 Scoring Analysis of Risk Genes

**FIGURE 4:**
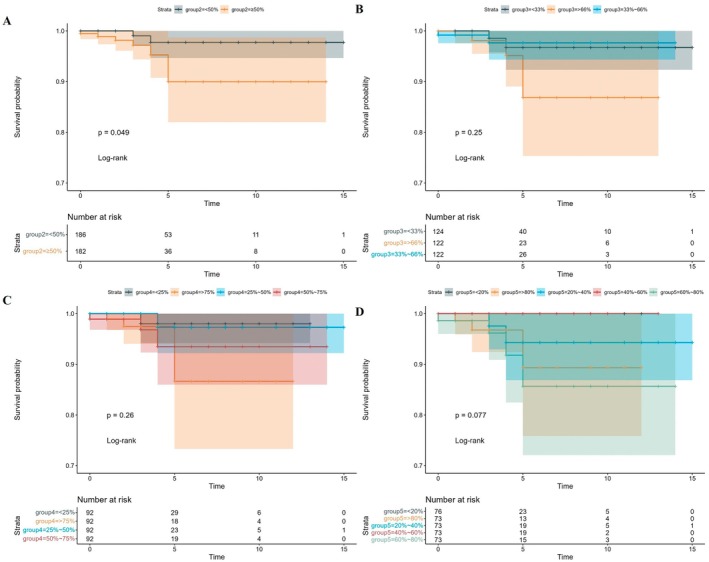
Kaplan–Meier (K‐M) analysis for different risk groups in the TCGA female Papillary Thyroid Cancer (PTC) cohort. Survival probabilities were estimated using the Kaplan–Meier method and compared using the log‐rank test. Patients were stratified into risk groups based on a risk score derived from the expression of F10, FOXD3, and SLC12A5. (A) Dichotomous survival analysis: Patients were divided into high‐risk (*n* = 182) and low‐risk (*n* = 186) groups based on the median risk score. The *p*‐value (*p* = 0.049) indicates a significant difference in overall survival between the two groups. (B) Three‐level survival analysis: Patients were divided into three groups based on tertiles of the risk score (group1: Lowest third, *n* = 124; group2: Middle third, *n* = 122; group3: Highest third, *n* = 122). (C) Four‐level survival analysis: Patients were divided into four groups based on quartiles of the risk score (group1: *N* = 92, group2: *N* = 92, group3: *N* = 92, group4: *N* = 92). (D) Five‐level survival analysis: Patients were divided into five groups based on quintiles of the risk score (group1: *N* = 76, group2: *N* = 73, group3: *N* = 73, group4: *N* = 73, group5: *N* = 73). The ‘Number at risk’ tables below each plot indicate the number of patients remaining at risk at various time points within each stratum.

We calculated a risk score for each individual based on the risk coefficient values obtained from multivariate Cox proportional hazards regression analysis. Using the median risk score as a cutoff, we classified the cohort into high‐risk (182 cases) and low‐risk (186 cases) groups. We observed that as patients' risk values increased, their risk scores and corresponding gene expression levels also rose (Figure 3E–G). Kaplan–Meier survival curve analysis indicated that mortality increased gradually over time, with the high‐risk group exhibiting significantly higher mortality after 5 years than the low‐risk group (*p* = 0.049). We then assigned patients to additional risk categories according to their risk values and confirmed that the dichotomous classification provided the most significant distinction (Figure 4).

To further investigate the association between additional factors and prognosis, we integrated age, tumor stage, T stage, and other relevant data. We then constructed univariate and multivariate Cox proportional hazards regression models to examine the correlation between risk score and clinical tumor stage. Our findings indicate that the risk score is an independent predictor of prognosis. Notably, the risk score exerted a stronger influence on patient outcomes than tumor T stage or age (Figure S1).

### 3.6 Infiltration Analysis of Risk Genes

To accurately assess the composition of immune cells in the tumor microenvironment, we performed immunoinfiltration analyses of the selected genes. The results indicated that the F10 gene was strongly correlated with CD8 + T cells, neutrophils, and dendritic cells, whereas FOXD3 was significantly associated with B cells, CD4 + T cells, neutrophils, macrophages, and dendritic cells. Additionally, SLC12A5 displayed moderate correlation with B cells, CD4 + T cells, neutrophils, and dendritic cells (Figure S2).

## 4 Discussion

In this cross‐sectional study, we identified a significant association between family history of hypertension and the prevalence of thyroid cancer among women at both genetic and clinical levels, whereas the association was less pronounced in men. Using integrated machine‐learning models, we found that women with a family history of hypertension had a higher risk of thyroid cancer. Moreover, our findings underscore the critical role of differentially expressed genes, particularly FOXD3, F10, and SLC12A5, in the pathogenesis of both hypertension and thyroid cancer. In recent years, in many regions worldwide, the incidence of thyroid cancer in women has been substantially higher than that in men, with the ratio reaching 3:1. This disparity highlights the importance of elucidating the pathogenesis and underlying mechanisms of thyroid cancer in women. Our study provides useful support for thyroid cancer control policies and offers insights into future research directions concerning the internal mechanisms of thyroid cancer.

Thyroid‐related disorders are strongly associated with various female‐specific conditions. Recent studies indicate a continuous rise in thyroid nodules during pregnancy, with a substantial proportion of women diagnosed with thyroid cancer during or shortly after pregnancy developing persistent or recurrent disease. Additionally, earlier research has investigated the link between hypertension and thyroid disease in pregnant women, demonstrating that subclinical hypothyroidism and dominant hyperthyroidism are associated with a higher risk of hypertensive outcomes such as pregnancy‐induced hypertension or preeclampsia [28, 29, 30]. Hence, it is especially important to examine the sex differences among individuals with similar characteristics. Our findings align with those of previous investigations. However, it is noteworthy that our study established a new connection between these conditions in a broader population that included non‐pregnant women, and we conducted a parallel analysis among men. The results revealed a strong association between family history of hypertension and an increased incidence of thyroid cancer, specifically in women. In contrast, no significant association was observed between family history of hypertension and the incidence of thyroid cancer in men.

In recent years, thyroid cancer prediction models have significantly improved in accuracy, driven by highly sensitive screening methods, such as high‐resolution ultrasound and MRI. However, current evidence suggests that these extremely precise imaging models often detect small tumors or lesions that are unlikely to reach clinical significance within a patient's lifetime, thereby contributing to the overtreatment of thyroid cancer. Being diagnosed with cancer imposes substantial psychological stress on patients and may lead to unnecessary medical interventions and associated side effects.

Consequently, there is a pressing need to re‐evaluate thyroid cancer screening strategies from epidemiological and big data perspectives, incorporating multifaceted patient information. In our study, we verified the role of family history of hypertension within an integrated model that included psychological factors and lifestyle habits and conducted a comprehensive analysis of each patient. We found that family history of hypertension holds considerable potential for individualized screening of thyroid cancer in women. Therefore, future research should consider incorporating family history of hypertension as a key predictor in machine learning models to enhance personalized thyroid cancer screening.

At the genetic level, our independent differential and prognostic analyses identified FOXD3, F10, and SLC12A5 as genes shared between thyroid cancer and hypertension. Previous studies have partially linked these three genes to tumor development. In particular, some studies have indicated that FOXD3 is associated with thyroid cancer risk [31, 32, 33, 34]. Moreover, it was shown to inhibit aggressive thyroid cancer behaviors by suppressing the lncRNA FOXD3‐AS1 by elevating miR‐296‐5p and inactivating the TGF‐β1/Smad signaling pathway, which aligns with the increased expression of FOXD3 observed in our study [35] SLC12A5, on the other hand, plays a critical role in tumor cell proliferation, metastasis, invasion, and apoptosis regulation. It is associated with tumor prognosis, MMRs, MSI, and TMB in various cancers including thyroid cancer [36, 37, 38]. SLC12A5 has been confirmed to be overexpressed in thyroid cancer, making it an important player in the progression of this disease [39]. F10, in contrast, is associated with lower expression in thyroid cancer patients, and reduced expression has been linked to an increased risk of disease recurrence [40]. Additionally, F10 is known to play a role in the regulation of other cancers [41, 42, 43], such as breast cancer [44, 45, 46]. Building on our previous study, we performed a separate prognostic analysis for female patients, and the results were consistent with the aforementioned findings for thyroid cancer. In addition, the genetic risk score derived from the differentially expressed genes confirmed that the high‐risk group exhibited significantly higher mortality after 5 years. This trend was not observed in men, which may explain the higher incidence of thyroid cancer in women. In light of these findings, incorporating family history of hypertension as a risk factor in thyroid cancer screening programs could significantly enhance early detection, particularly in high‐risk women. Genetic markers, such as FOXD3 and SLC12A5, may serve as potential diagnostic biomarkers, facilitating more precise, personalized screening strategies for thyroid cancer.

In addition, we found that the expression profiles of FOXD3, F10, and SLC12A5 in immunoassays correlated with the levels of immune cells, neutrophils, and dendritic cells in tumor tissues. The role of these three genes and their corresponding models in prognosis can be further analyzed from an immunological perspective in future research, as the involvement of immune‐related genes suggests that targeted therapies could regulate the tumor microenvironment. For example, approaches that enhance CD4 + T cell activity may introduce new strategies to improve the immune response in patients with thyroid cancer. In summary, the overlapping genes related to both thyroid cancer and hypertension warrant further investigation as potential therapeutic targets.

Furthermore, our findings regarding the association between a family history of hypertension and increased thyroid cancer risk in women may be contextualized within the broader impact of metabolic dysregulation, such as Metabolic Syndrome (MetS) and Insulin Resistance Syndrome (IRS), on oncogenesis [47, 48]. IRS is recognized for fostering a systemic chronic low‐grade inflammatory state and immune dysfunction. This condition is characterized by elevated pro‐inflammatory cytokines (e.g., TNF‐α, IL‐6), dysregulated adipokines like leptin and adiponectin, and aberrant immune cell activity, including altered macrophage polarization and T‐cell function. Such an IRS‐driven pro‐inflammatory environment is implicated in the development and progression of various malignancies, including but not limited to colorectal, breast, and pancreatic cancers, by impacting crucial cellular processes such as proliferation, survival, angiogenesis, and immune evasion [49].

Chronic inflammation and immune dysregulation driven by IRS have been implicated in the pathogenesis of colorectal, breast, pancreatic, and liver cancers, with underlying mechanisms involving aberrant insulin/IGF‐1 signaling and sustained activation of proinflammatory pathways such as PI3K/Akt/mTOR and NF‐κB. Notably, the genes identified in our study—FOXD3, F10, and SLC12A5—may also participate in these IRS‐mediated inflammatory pathways in other tumor types. For example, FOXD3 is involved in TGF‐β signaling regulation and pancreatic β‐cell function; F10 links coagulation to inflammation via activation of the protease‐activated receptor‐2 (PAR‐2) pathway; and SLC12A5 has been reported to induce endoplasmic reticulum stress and activate the PI3K/Akt/mTOR cascade. Further investigation into the precise roles of these genes in metabolically related cancers may yield novel insights for risk assessment and preventive strategies across diverse malignancies [50, 51, 52].

This study has several limitations. Our data collection was conducted primarily in China, which may restrict the external generalizability of our findings. Furthermore, this dataset includes an uneven distribution of thyroid cancer cases. However, by amplifying the data and integrating multiple models based on this, we have reduced the imbalance while enhancing the authenticity of the data. A key limitation of our genomic analysis is the multi‐ethnic composition of the TCGA cohort and the lack of race‐stratified evaluation. As our clinical cohort was drawn exclusively from a Chinese population, these differences may affect the applicability of our genomic findings across diverse racial groups. Moreover, a limitation in our TCGA genomic analysis is the absence of explicit correction for potential batch effects. Unaddressed batch effects from varied sample processing could influence the identified differentially expressed genes. Future work should include thorough assessment and, if necessary, correction of such technical confounders to enhance the robustness of genomic findings.

## 5 Conclusions

The results underscored a significant association between family history of hypertension and thyroid cancer in women, which did not appear in men. Future research should investigate the distinct mechanisms underlying hypertension and thyroid cancer in women to attain a more comprehensive understanding of their causes. Moreover, our study offers new directions for addressing the rising incidence of thyroid cancer in women, and may further optimize screening and prevention programs.

## Author Contributions

**Zijin Wang:** conceptualization, resources, writing – original draft, investigation, methodology. **Yanhui Lin:** conceptualization, resources, data curation. **Fanke Meng:** writing – review and editing, supervision, conceptualization, investigation. **Yuxin Sun:** conceptualization, validation, software. **Ziran Zhang:** investigation, methodology, visualization. **Tong Wu:** validation, visualization, writing – original draft. **Min Fu:** writing – review and editing, conceptualization, validation, methodology. **Fanye Wu:** software, methodology, visualization, writing – original draft. **Zhengran Li:** data curation, conceptualization, methodology. **Zejun Chen:** conceptualization, visualization, writing – original draft.

## Disclosure

*Declaration of generative AI and AI‐assisted technologies in the writing process*: During the preparation of this manuscript, the first author used ChatGPT (Scholar) as an editing assistant to refine the language. All suggestions provided by the AI were carefully reviewed and selectively incorporated. The authors affirm that they take full responsibility for the content of this publication.

## Ethics Statement

Data for this cross‐sectional study were obtained from the Third Xiangya Hospital of Central South University. Informed written consent was obtained from all participants, and the study protocol was approved by the Ethics Committee of the Third Xiangya Hospital of Central South University (No. 22206).

## Conflicts of Interest

The authors declare no conflicts of interest.

## Supporting information


**Data S1.** Supporting Information.

## Data Availability

The datasets generated and/or analyzed during the study are available from the corresponding author upon reasonable request.
