# Supplementary material for: Family History of Hypertension Predicts Thyroid Cancer Risk in Women: A Population‐Based Cross‐Sectional Study With Integrative Machine Learning and Genomic Analyses
Source: Cancer Med. 2025 Aug 10;14(15):e71031. doi: 10.1002/cam4.71031 (PMC12336287; doi:10.1002/cam4.71031)
Supplement: Supplementary file 1 — Data S1. Supporting Information. [file CAM4-14-e71031-s001.docx]

**Supplementary Online Content**

**eTable 1.** Psychological quantification scale

**eTable 2.** Descriptive statistics of selected continuous features in the training set before and after SMOTENC application

**eTable 3.** Hyperparameter grids used for the optimization of base machine learning models

**eTable 4.** Differential variable analysis of thyroid cancer in women

**eTable 5.** Forward and backward feature screening by logistic regression in women

**eTable 6.** Binary logistic regression results for men

**eTable 7.** Results of univariate Cox proportional-hazards model

**eFigure 1.** Association with T-stage and STAGE

eFigure 2. Analysis of single gene infiltration in six immune cell types

eTable 1: Psychological quantification scale

| Characteristics of mental health | No | Sometimes | Always |
| --- | --- | --- | --- |
| Sullen and unhappy | 33967 | 17387 | 1709 |
| Excited and easily upset | 29876 | 20326 | 2861 |
| Tension that makes it difficult to relax | 35597 | 15070 | 2396 |
| More tense and anxious than usual | 37416 | 13439 | 2208 |
| Tantrums and impatience | 31732 | 18282 | 3049 |
| Drained and unenthusiastic | 37481 | 13754 | 1828 |
| Anxious and distracted | 36548 | 14524 | 1991 |
| Depressed and frustrated | 38122 | 13192 | 1749 |
| Difficulty concentrating | 37943 | 13813 | 1307 |

eTable 2: Descriptive statistics of selected continuous features in the training set before and after SMOTENC application

| Characteristic | Class | Before SMOTE | | | | | After SMOTE | | | | |
| --- | --- | --- | --- | --- | --- | --- | --- | --- | --- | --- | --- |
|  |  | Mean | SD | 25% | 50% | 75% | Mean | SD | 25% | 50% | 75% |
| BMI | Total | 22.47 | 2.96 | 20.42 | 22.13 | 24.13 | 22.53 | 2.97 | 20.46 | 22.19 | 24.18 |
| BMI | HC | 22.46 | 2.96 | 20.41 | 22.12 | 24.13 | 22.46 | 2.96 | 20.41 | 22.12 | 24.13 |
| BMI | THCA | 23.08 | 3.15 | 21.03 | 22.70 | 24.54 | 23.13 | 2.96 | 21.09 | 22.86 | 24.58 |
| WC | Total | 74.10 | 8.15 | 68.00 | 73.00 | 79.00 | 74.14 | 8.11 | 68.00 | 73.00 | 79.00 |
| WC | HC | 74.09 | 8.15 | 68.00 | 73.00 | 79.00 | 74.09 | 8.15 | 68.00 | 73.00 | 79.00 |
| WC | THCA | 75.04 | 8.12 | 69.00 | 74.00 | 80.00 | 74.58 | 7.70 | 69.00 | 74.00 | 79.00 |
| TG | Total | 1.20 | 0.87 | 0.72 | 0.98 | 1.41 | 1.20 | 0.85 | 0.72 | 0.99 | 1.42 |
| TG | HC | 1.20 | 0.87 | 0.72 | 0.98 | 1.41 | 1.20 | 0.87 | 0.72 | 0.98 | 1.41 |
| TG | THCA | 1.20 | 0.70 | 0.69 | 1.02 | 1.42 | 1.21 | 0.57 | 0.78 | 1.12 | 1.50 |
| HDL | Total | 1.44 | 0.27 | 1.25 | 1.42 | 1.61 | 1.44 | 0.27 | 1.26 | 1.42 | 1.61 |
| HDL | HC | 1.44 | 0.27 | 1.25 | 1.42 | 1.61 | 1.44 | 0.27 | 1.25 | 1.42 | 1.61 |
| HDL | THCA | 1.46 | 0.26 | 1.28 | 1.42 | 1.62 | 1.44 | 0.21 | 1.29 | 1.43 | 1.60 |
| Weight | Total | 56.26 | 7.88 | 50.80 | 55.30 | 60.60 | 56.43 | 7.90 | 51.00 | 55.50 | 60.90 |
| Weight | HC | 56.24 | 7.88 | 50.80 | 55.30 | 60.60 | 56.24 | 7.88 | 50.80 | 55.30 | 60.60 |
| Weight | THCA | 58.36 | 8.22 | 52.93 | 57.65 | 62.53 | 58.36 | 7.89 | 53.06 | 57.90 | 62.58 |
| Height | Total | 158.24 | 5.45 | 154.50 | 158.00 | 162.00 | 158.29 | 5.33 | 155.00 | 158.00 | 162.00 |
| Height | HC | 158.23 | 5.45 | 154.50 | 158.00 | 162.00 | 158.23 | 5.45 | 154.50 | 158.00 | 162.00 |
| Height | THCA | 159.02 | 4.70 | 155.63 | 158.75 | 162.00 | 158.82 | 3.87 | 156.14 | 158.87 | 161.48 |

Abbreviation: Standard Deviation (SD); High-Density Lipoprotein Cholesterol (HDL); Body Mass Index (BMI); Healthy Control (HC); Thyroid Cancer (THCA); Synthetic Minority Oversampling Technique (SMOTE); Waist Circumference (WC); Triglycerides (TG)

eTable 3: Hyperparameter grids used for the optimization of base machine learning models

| Model Type | Hyperparameter | Searched Values/Ranges |
| --- | --- | --- |
| XGBoost | max_depth | 3, 5, 7 |
| XGBoost | n_estimators | 200, 150, 100 |
| XGBoost | learning_rate | 0.001, 0.01, 0.1 |
| HistGBDT | max_depth | 3, 5, 7 |
| HistGBDT | learning_rate | 0.001, 0.01, 0.1 |
| CatBoost | depth | 5, 6, 7 |
| CatBoost | iterations | 300 |
| Decision Tree | max_depth | 3, 5, 7 |
| Logistic Regression | C | 0.01, 0.1, 1, 5, 10 |
| Logistic Regression | solver | liblinear, saga |
| Model Type | Hyperparameter | Searched Values/Ranges |

eTable 4: Differential variable analysis of thyroid cancer in women

| **Characteristic** | **Female with thyroid cancer (n=204), (n, %)** | **Controls (n=25635), (n, %)** | **P value** |
| --- | --- | --- | --- |
| Family history |  |  |  |
| Other | 94 (46.08) | 15457 (60.30) | <0.001 |
| Hypertension | 70 (34.31) | 8001 (31.21) |  |
| Cancer | 40 (19.61) | 2177 (8.49) |  |
| Alcohol Consumption |  |  |  |
| Never or Former | 198 (97.06) | 23977 (93.53) | 0.041 |
| Active | 6 (2.94) | 1658 (6.47) |  |
| Exercise status |  |  |  |
| Seldom | 66 (32.35) | 10429 (40.68) | 0.016 |
| Frequently | 138 (67.65) | 15206 (59.32) |  |
| Drained and unenthusiastic |  |  |  |
| Never | 154 (75.49) | 17592 (68.62) | 0.50 |
| Sometimes | 43 (21.08) | 6943 (27.08) |  |
| Always | 7 (3.43) | 1100 (4.29) |  |
| Depressed and frustrated |  |  |  |
| Never | 156 (76.47) | 17645 (68.83) | 0.06 |
| Sometimes | 39 (19.12) | 6927 (27.02) |  |
| Always | 9 (4.41) | 1063 (4. 15) |  |
| Anxious and distracted |  |  |  |
| Never | 145 (71.08) | 16770 (65.42) | 0.211 |
| Sometimes | 49 (24.02) | 7633 (29.78) |  |
| Always | 10 (4.90) | 1232 (4.81) |  |
| Exercise Duration (hours) | 1.33 (1.04) [0.00 - 3.00] | 1.09 (1.03) [0.00 - 3.00] | 0.001 |
| Exercise frequency (times/week) | 1.78 (1.72) [0.00 - 5.00] | 1.43 (1.62) [0.00 - 5.00] | 0.002 |
| Exercise History (years) | 1.40 (1.24) [0.00 - 4.00] | 1.21 (1.25) [0.00 - 4.00] | 0.016 |
| Height (cm) | 159.31 (5.07) [145.50 - 172.50] | 158.21 (5.46) [133.50 - 181.00] | 0.006 |
| Weight | 58.51 (8.32) [41.50 - 102.00] | 56.23 (7.87) [31.90 - 119.00] | <0.001 |
| BMI | 23.04 (2.92) [16.94 - 34.48] | 22.47 (2.97) [13.99 - 45.75] | 0.002 |
| Hip Circumference (cm) | 93.35 (5.73) [81.00 - 117.00] | 92.28 (5.38) [70.00 - 130.00] | 0.006 |
| Waist Circumference (cm) | 75.01 (7.97) [57.00 - 113.00] | 74.11 (8.16) [52.00 - 128.00] | 0.074 |
| DBP (MmHg) | 71.14 (9.26) [52.00 - 98.00] | 69.87 (9.77) [41.00 - 121.00] | 0.028 |
| SBP (MmHg) | 117.34 (12.91) [90.00 - 154.00] | 116.52 (14.94) [67.00 - 214.00] | 0.075 |

Abbreviation: Systolic Blood Pressure (SBP); Diastolic Blood Pressure (DBP); Body Mass Index (BMI)

**eTable 5: Forward and backward feature screening by logistic regression in women**

| **Characteristic** | **Female with thyroid cancer (n=204), (n, %)** | **Controls (n=25635), (n, %)** | **OR (95% CI)** | **P value** |  |
| --- | --- | --- | --- | --- | --- |
| **Forward** |  |  |  |  |  |
| Family History |  |  |  |  |  |
| Other | 94 (46.08) | 15457 (60.3) | 1 [Reference] | NA |  |
| Hypertension | 70 (34.31) | 8001 (31.21) | 1·53 (1·09-2·14) | ·04 |  |
| Cancer | 40 (19.61) | 2177 (8.49) | 3.0 (2.06 - 4.35) | <·001 |  |
| Alcohol Consumption |  |  |  |  |  |
| Never or Former | 198 (97.06) | 23977 (93.53) | 1 [Reference] | NA |  |
| Active | 6 (2.94) | 1658 (6.47) | 0.41 (0.18 - 0.92) | ·03 |  |
| Depressed and frustrated |  |  |  |  |  |
| Never | 156 (76.47) | 17645 (68.83) | 1 [Reference] | NA |  |
| Sometimes | 39 (19.12) | 6927 (27.02) | 0.66 (0.46-0.94) | .02 |  |
| Always | 9 (4.41) | 1063 (4. 15) | NA | >0.05 |  |
| Weight (kg) |  |  | 1.07 (1.04 - 1.10) | <·001 |  |
| Mean (SD) [range] | 58.51 (8.32), [41.50-102.0] | 56.23 (7.87), [31.90-119.0] |  |  |  |
| Median (IQR) | 58.00 (52.88-62.75) | 55.30 (50.90-60.50) |  |  |  |
| Exercise Duration (hours) |  |  | 1.24 (1.09 - 1.42) | ·002 |  |
| Mean (SD) [range] | 1.33 (1.04), [0.0-3.0] | 1.09 (1.03), [0.0-3.0] |  |  |  |
| Median (IQR) | 2.0 (0.0-2.0) | 1.0 (0.0-2.0) |  |  |  |
| Waist Circumference (cm) |  |  | 0.96 (0.93 - 0.99) | ·005 |  |
| Mean (SD) [range] | 75.01 (7.97), [57.0-113.0] | 74.11 (8.16) [52.0-128.0] |  |  |  |
| Median (IQR) | 74.0 (69.0-79.0) | 73.0 (68.0-79.0) |  |  |  |
| **Backward** |  |  |  |  |  |
| Family History |  |  |  |  |  |
| Other | | 94 (46.08) | 15457 (60.3) | 1 [Reference] | NA |
| Hypertension | | 70 (34.31) | 8001 (31.21) | 1.37 (1.0-1.88) | 0·05 |
| Cancer | | 40 (19.61) | 2177 (8.49) | 3.06 (2.11-4.45) | <0·001 |
| Alcohol Consumption | |  |  |  |  |
| Never or Former | | 198 (97.06) | 23977 (93.53) | 1 [Reference] | NA |
| Active | | 6 (2.94) | 1658 (6.47) | 0.42 (0.18-0.94) | 0·04 |
| Depressed and frustrated | |  |  |  |  |
| Never | | 156 (76.47) | 17645 (68.83) | 1 [Reference] | NA |
| Sometimes | | 39 (19.12) | 6927 (27.02) | 0.66 (0.46-0.94) | 0.02 |
| Always | | 9 (4.41) | 1063 (4. 15) | NA | >0.05 |
| Weight (kg) | |  |  | 1.07 (1.04 - 1.10) | <0·001 |
| Mean (SD) [range] | | 58.51 (8.32), [41.50-102.0] | 56.23 (7.87), [31.90-119.0] |  |  |
| Median (IQR) | | 58.0 (52.88-62.75) | 55.30 (50.90-60.50) |  |  |
| Exercise Duration (hours) | |  |  | 1.24 (1.08-1.41) | 0·002 |
| Mean (SD) [range] | | 1.33 (1.04), [0.0-3.0] | 1.09 (1.03), [0.0-3.0] |  |  |
| Median (IQR) | | 2.0 (0.0-2.0) | 1.0 (0.0-2.0) |  |  |
| Waist Circumference (cm) | |  |  | 0.95 (0.92-0.98) | 0·002 |
| Mean (SD) [range] | | 75.01 (7.97), [57.0-113.0] | 74.11 (8.16) [52.0-128.0] |  |  |
| Median (IQR) | | 74.0 (69.0-79.0) | 73.0 (68.0-79.0) |  |  |
| LDL (Mmol/L) | |  |  | 0.25 (0.08-0.75) | 0.01 |
| Mean (SD) [range] | | 2.87 (0.80) [0.88 - 5.20] | 2.92 (0.79) [0.36 - 10.83] |  |  |
| Median (IQR) | | 2.90 (2.30-3.31) | 2.84 (2.37-3.38) |  |  |
| HDL (Mmol/L) | |  |  | 0.22 (0.06-0.78) | 0.02 |
| Mean (SD) [range] | | 1.42 (0.26) [0.89 - 2.45] | 1.44 (0.27) [0.52 - 3.06] |  |  |
| Median (IQR) | | 1.40 (1.23-1.58) | 1.42 (1.25-1.61) |  |  |
| Total Cholesterol (Mmol/L) | |  |  | 3.56 (1.23-10.31) | 0.02 |
| Mean (SD) [range] | | 4.88 (0.94) [2.44 - 7.49] | 4.92 (0.95) [1.95 - 14.99] |  |  |
| Median (IQR) | | 4.88 (4.21-5.44) | 4.82 (4.25-5.48) |  |  |
| Triglycerides (Mmol/L) | |  |  | 0.62 (0.38-1.0) | 0.05 |
| Mean (SD) [range] | | 1.25 (0.80) [0.22 - 4.92] | 1.21 (0.92) [0.19 - 23.06] |  |  |
| Median (IQR) | | 1.02 (0.73-1.51) | 0.98 (0.72-1.42) |  |  |

Abbreviation: high density lipoprotein (HDL); low density lipoprotein (LDL); odds ratio (OR); confidence interval (CI); standard deviation (SD) and interquartile range (IQR)

eTable 6: Binary logistic regression results for men

| **Characteristics** | **Male patients with thyroid cancer, (n=92) (n, %)** | **Controls, (n=27157) (n, %)** | **OR (95% CI)** | **P value** | **P for trend** |
| --- | --- | --- | --- | --- | --- |
| Family history |  |  |  |  |  |
| Other | 35(38.04) | 15196(55.96) | 1 [Reference] | NA | <0.001 |
| Hypertension | 23 (25.00) | 7240 (26.66) | 1.306 (0.770-2.214) | 0.32 |  |
| Coronary heart disease | 8 (8.70) | 1944 (7.1.58) | 1.699 (0.786-3.674) | 0.18 |  |
| Diabetes mellitus | 5 (5.43) | 758 (2.79) | 2.716 (1.058-6.975) | 0.04 |  |
| Cancer | 21 (22.83) | 2019(7.44) | 4.348 (2.522-7.499) | <0.001 |  |
| Alcohol consumption |  |  |  |  |  |
| Never drinkers | 65 (70.65) | 14381 (52.96) | 1 [Reference] | NA | 0.009 |
| Active | 24 (26.09) | 12224 (45.01) | 0.475 (0.293-0.772) | 0.003 |  |
| Former | 3 (3.26) | 552 (2.03) | 1.155 (0.356-3.749) | 0.81 |  |
| Smoking status |  |  |  |  |  |
| Never smokers | 57 (61.96) | 14483 (53.33) | 1 [Reference] | NA | 0.04 |
| Passive | 2 (2.17) | 1283 (4.72) | 0.475 (0.115-1.960) | 0.30 |  |
| Former | 11 (11.96) | 1425 (5.25) | 2.018 (1.043-3.903) | 0.04 |  |
| Active | 22 (23.91) | 9966 (36.70) | 0.730 (0.439-1.213) | 0.22 |  |
| Exercise history (years) |  |  |  |  |  |
| No | 23 (25.00) | 9033 (33.26) | 1 [Reference] | NA | 0.045 |
| <1 | 20 (21.74) | 3813 (14.04) | 1.732 (0.946-3.171) | 0.08 |  |
| 1-5 | 33 (35.87) | 7006 (25.80) | 1.604 (0.934-2.757) | 0.09 |  |
| 6-10 | 4 (4.35) | 2848 (10.49) | 0.489 (0.168-1.425) | 0.19 |  |
| >10 | 12 (13.04) | 4457 (16.41) | 0.931 (0.458-1.892) | 0.84 |  |
| Hip circumference (cm) |  |  |  |  |  |
| Mean (SD) [range] | 98.28 (6.07) [83-120] | 96.79 (5.64) [63-132] | 1.051 (1.015-1.089) | 0.005 | NA |
| Median (IQR) | 97 (8) | 97 (7) |  |  |  |

Abbreviations: odds ratio (OR); confidence interval (CI); standard deviation (SD) and interquartile range (IQR)

eTable 7: Results of univariate Cox proportional-hazards model

| **Genes** | **HR** | **P value** |
| --- | --- | --- |
| APOD | 1.00 | <.01 |
| BIRC7 | 1.00 | <.01 |
| CAPN8 | 1.00 | <.01 |
| CD177 | 1.00 | <.01 |
| CDKN2A | 1.00 | <.01 |
| CFI | 1.00 | .01 |
| CHST6 | 1.00 | <.01 |
| CNGA3 | 1.01 | <.01 |
| CSF2 | 1.00 | <.01 |
| CTSC | 1.00 | .02 |
| DOK7 | 1.00 | .03 |
| DUXAP8 | 1.00 | <.01 |
| *F10* | 1.01 | <.01 |
| *FOXD3* | 1.14 | <.01 |
| GLI1 | 1.01 | <.01 |
| GRIA1 | 1.07 | .04 |
| IBSP | 1.00 | <.01 |
| IFNA1 | 1.03 | <.01 |
| IGHE | 1.00 | .02 |
| KISS1R | 1.01 | .03 |
| LGI1 | 1.12 | .01 |
| MDK | 1.00 | <.01 |
| MUSK | 1.02 | .04 |
| MYH2 | 1.00 | <.01 |
| NAPSA | 1.00 | <.01 |
| OGN | 1.00 | .02 |
| PCSK1N | 1.00 | <.01 |
| PCSK9 | 1.02 | <.01 |
| POSTN | 1.00 | <.01 |
| PRSS1 | 1.00 | <.01 |
| RETN | 1.00 | <.01 |
| SHH | 1.00 | <.01 |
| *SLC12A5* | 1.00 | <.01 |
| SLC1A7 | 1.01 | .04 |
| STRA6 | 1.00 | <.01 |
| TCL6 | 1.01 | <.01 |
| TNNI3 | 1.00 | <.01 |
| TNNT1 | 1.00 | <.01 |
| TNNT3 | 1.00 | <.01 |
| VCAN | 1.00 | <.01 |
| XDH | 1.00 | .04 |

Abbreviations: hazard ratio (HR)


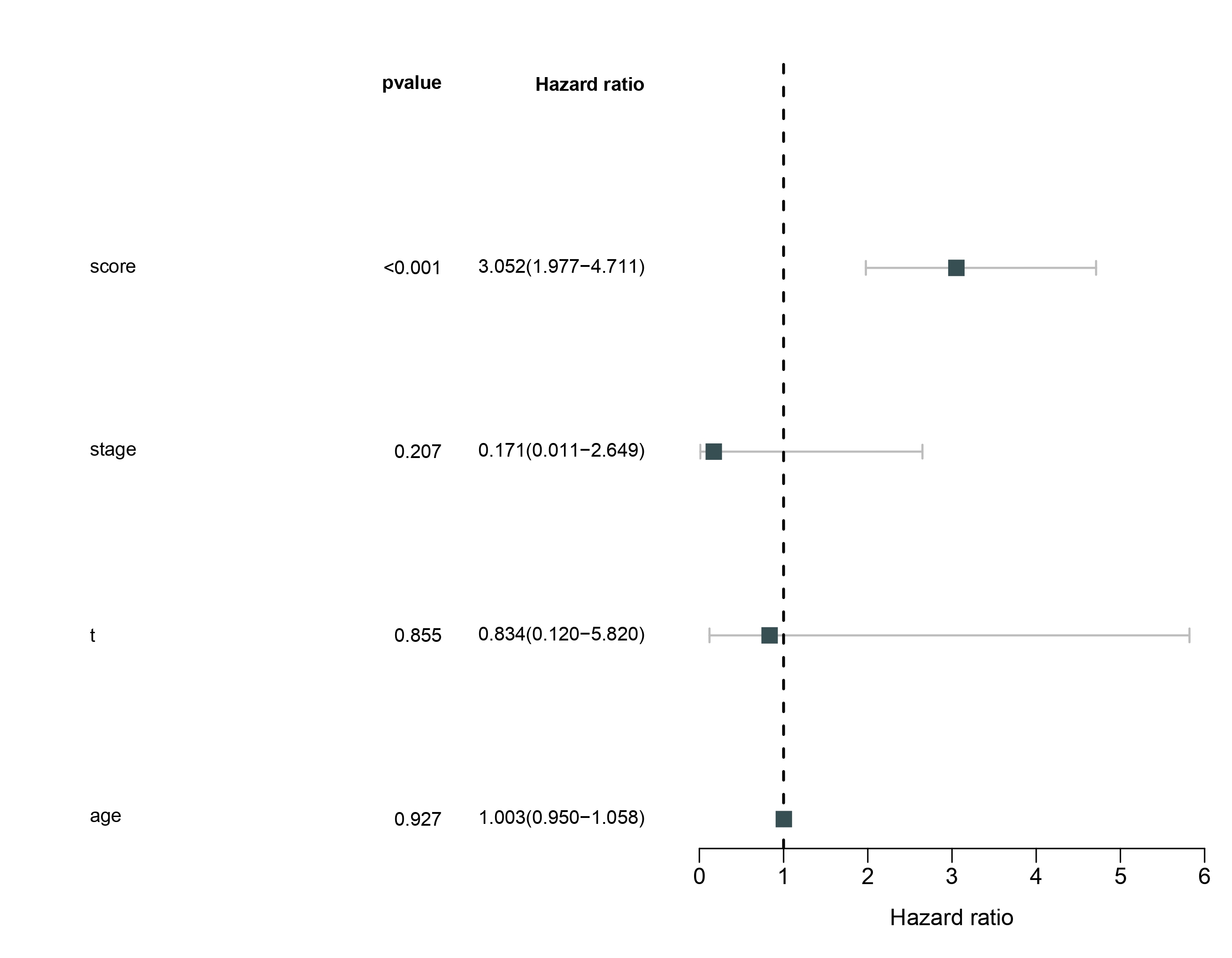


eFigure 1: Forest plot of clinical factors and risk score

^a^ The stage factor is based on the latest American Joint Committee on Cancer (AJCC) tumour staging method.

^b^ This refers to the condition of the primary tumour, which increases with tumour volume and the extent of adjacent tissue involvement, and is denoted by T1 to T4 in that order. T0 indicates no evidence of a primary tumour, and T1-T4 indicates an increase in tumour size and impact on surrounding tissues as the primary tumour increases, where we classify T1 and T2 as group 1, and T3 and T4 as group 2.


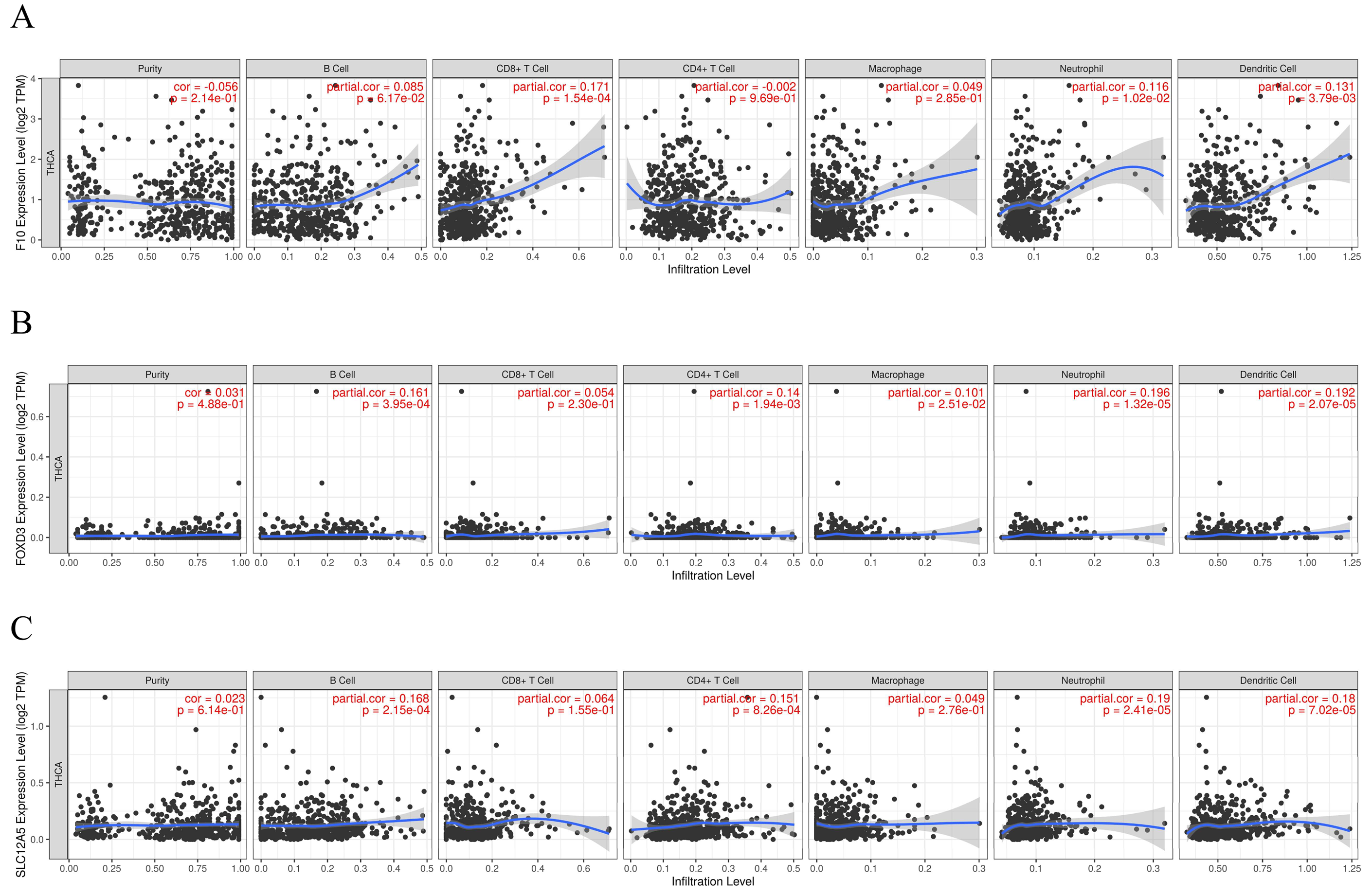


eFigure 2: Analysis of single gene infiltration in six immune cell types
